# Heart Rate Variability Analysis to Evaluate Autonomic Nervous System Maturation in Neonates: An Expert Opinion

**DOI:** 10.3389/fped.2022.860145

**Published:** 2022-04-21

**Authors:** Hugues Patural, Patricia Franco, Vincent Pichot, Antoine Giraud

**Affiliations:** ^1^Neonatal and Pediatric Intensive Care Department, Centre Hospitalier Universitaire de Saint-Étienne, Saint-Étienne, France; ^2^INSERM, U1059, SAINBIOSE, Université de Lyon, Université Jean-Monnet, Saint-Étienne, France; ^3^Sleep and Neurological Functional Explorations, Hôpital Femme Mère Enfant, Hospices Civils de Lyon, Lyon, France

**Keywords:** autonomic nervous system, sudden infant death syndrome (SIDS), life-threatening events, neonate, cardiac monitoring

## Abstract

While heart rate variability (HRV) is a relevant non-invasive tool to assess the autonomic nervous system (ANS) functioning with recognized diagnostic and therapeutic implications, the lack of knowledge on its interest in neonatal medicine is certain. This review aims to briefly describe the algorithms used to decompose variations in the length of the RR interval and better understand the physiological autonomic maturation data of the newborn. Assessing newborns’ autonomous reactivity can identify dysautonomia situations and discriminate children with a high risk of life-threatening events, which should benefit from cardiorespiratory monitoring at home. Targeted monitoring of HRV should provide an objective reflection of the newborn’s intrinsic capacity for cardiorespiratory self-regulation.

## Introduction

Like adult pathology (1), the impact of autonomic nervous system (ANS) dysfunctions on children’s health is well established. Regardless of age (2), heart rate variability analysis (HRV) is a relevant non-invasive tool of real-time or delayed evaluation of autonomic function with recognized diagnostic and therapeutic implications (3–8). Measurement tools that consider variations in the length of the RR interval, beat after beat, are widely available, and reference values according to the child’s age have been published (9, 10).

This narrative review aims to overview the various HRV analysis techniques to evaluate autonomic nervous system maturation in neonates. We will also discuss the potential implications of ANS maturation studies to prevent sudden infant death syndrome and guide cardiac monitoring in neonatology units.

## Generalities About Cardiac Signal Processing

Analysis of HRV obtained from the heart electrical signal by a monitor connected to two or three thoracic electrodes can be carried out offline (e.g., from a cardiac Holter) or in real-time from sliding windows analyzing cardiac irregularity according to a sampling frequency between 200 and 1000 Hz (11–13).

**FIGURE 1:**
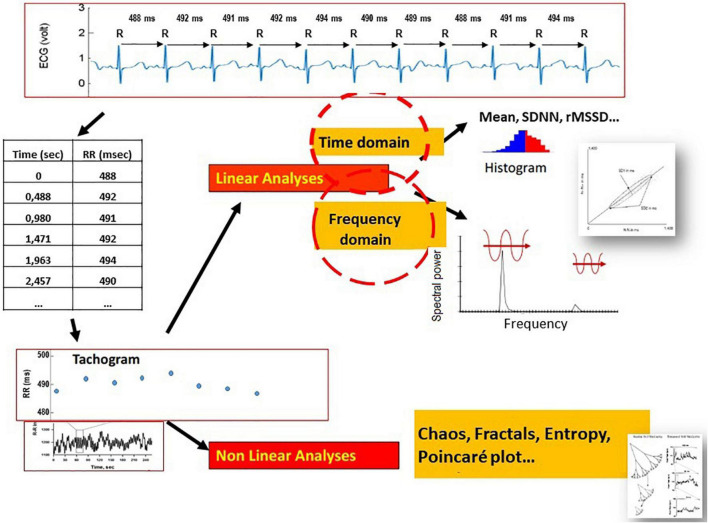
Electrocardiographic signal decomposition and beat-to-beat measurement (ms) of the RR suite to obtain a spectrogram over a given period, processed by mathematical models adapted to linear sequences (time domain, frequency domain) or non-linear sequences (chaos, fractal, entropy, and Poincaré plot).

A series of R-R intervals with an accuracy from 1 to 5 ms is generated from each detected R peak. Missing or ectopic beats and artifacts are corrected using cubic interpolations (12). The curve of these intervals (tachogram) is then processed by algorithms (Figure 1).

### Linear Analysis

In this configuration, signal analysis conventionally relies on the Fast Fourier Transform (FFT), method to assess the different frequencies in the RR series which requires the acquisition of stationary data with signal stability during the sampling period (13).

### Time-Domain Analysis (12)

It is based on the means and standard deviations measurements of the short and long-term variations of the RR intervals. The standard deviation of the RR intervals (SDNN), the standard deviation of the mean of all RR intervals for 5-minute segments (SDANN), and the mean of the standard deviation of all 5-minute RR intervals (SDNNIDX) represent long-term global variations. The percentage difference between adjacent normal RR intervals greater than 50 msec (pNN50) and the square root of the mean of the sum of the differences between normal RR intervals squared (rMSSD) represent the rapid changes associated with the parasympathetic activity. The geometric indices calculated on the density distribution of the RR intervals correspond to the assignment of the number of RR intervals of the same length to each value of their length.

The Poincaré plot is a scatter plot developed by plotting each RR interval against the previous one. It is analyzed quantitatively by fitting an ellipse whose shape is plotted with the average RR interval as the ellipse’s center. SD1 (short-term variability) represents the standard deviation of the Poincaré plot perpendicular to the identity line. In contrast, SD2 (long-term variability) means the standard deviation of the plot along this identity line.

### Frequency Domain Analysis (12)

A frequency spectrum from 0 to 2 Hz segmented into three main bands of interest as standardized by the Task Force in 1996 (11) and defines the regulation of the human cardiac signal: very low frequencies (VLF) from 0 to 0.04 Hz reflect the long-term regulatory mechanisms (thermoregulation, vasomotor tone peripheral, renin-angiotensin system), low frequencies (LF) from 0.04 to 0.15 Hz correspond to the involvement of mainly the sympathetic system and more incidentally of the parasympathetic system, and high frequencies (HF) of 0.15 to 2 Hz in newborns correspond to the ventilatory component under the exclusive control of the parasympathetic system. Total power (Ptot) represents overall variability. Normalized indices (LFnu, HFnu) and LF/HF ratio estimate sympathetic modulation and autonomic balance.

### Geometric Analysis (12)

This analysis defines the triangular index (integral of the density distribution divided by the maximum of the density distribution) and the TINN index (triangular interpolation of the RR interval histogram, i.e., the width of the base of this triangle). These measurements quantify the overall HRV primarily influenced by slow oscillations of the RR intervals.

### Non-linear Analysis (12)

Transition periods are evaluated by segmentation of the signal with the wavelet transform method (14), allowing better evaluation of non-stationary signals and more refined real-time analysis. The indices resulting from this approach provide information on the complexity of autonomic regulations. We can distinguish fractal values, which quantify the repetition of the patterns displayed at different scales. Fractal values are based on trend fluctuation analysis (α1, α2, H), slope (1/f), exponent (Hurst, Higuchi, Katz, Lyapunov). Entropy values can also estimate the regularity and complexity of a pattern over different lengths (entropy indices of Shanon and its derivatives, conditional entropy, sampled and approximated entropy).

Another non-linear approach consists in measuring the deceleration (DC) and acceleration (AC) capacities of two successive RR beat sequences to estimate the vagal and sympathetic powers.

### Cardiorespiratory Coupling

Other approaches to autonomic steady state analysis incorporate the link of instantaneous fluctuations between heart and respiratory rates over time using wavelet transforms. This is the cardiorespiratory coherence whose most significant reflection is represented by the physiological sinus arrhythmia caused by the respiratory cycle in the full-term baby with a healthy heart. In this case, if the child inhales and exhales, the HR increases and decreases in synchrony. In a situation of physiological stress, this coupling between heart rate and respiration could be attenuated. However, respiratory immaturity and the severity of central apneas are inversely correlated with gestational age and current treatment strategies based on caffeine and non-invasive respiratory assistance make it possible to overcome the initial stage of immature breathing. So when the full term approaches, the cardiorespiratory coupling is usually efficient. Currently the analysis of the cardiorespiratory coherence is not routinely used to guide monitoring for discharge but mainly concerns anesthesia and the perioperative period and proves to be of interest for evaluating nociception (15–17).

## Physiological Autonomic Maturation

In neonatal medicine, understanding vital physiological systems during the first months of life must integrate the notion of autonomic control system maturation. Thus *in utero*, it has been established that at least 37 weeks of maturation are necessary to achieve complete autonomous maturation at birth, particularly the parasympathetic system (2, 17, 18).

**FIGURE 2:**
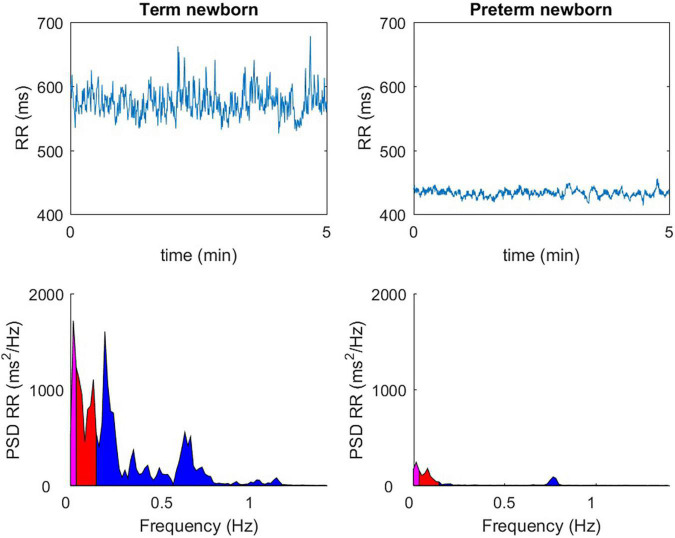
The observable difference over 5 min in quiet sleep, between a full-term newborn (40 wGA) and a premature newborn (36 wGA), with a temporal analysis (ms) of the RR spaces (top windows), or with a frequency domain representation (ms2 / Hz) (bottom windows).

For premature newborns regardless of gestational age (GA)(19, 20)and including late prematurity (21), cardiac reactivity, and the baroreflex loop are altered at theoretical term compared to term newborns (22, 23) (Figure 2), even if with postnatal age there is a significant increase in HRV parameters, in particular for the high-frequency index (HF), recognized as a relevant indicator of parasympathetic maturation.

**FIGURE 3:**
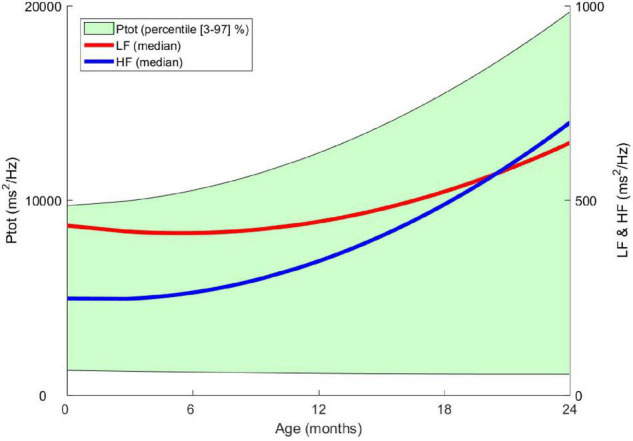
From birth to the age of 2 years, evolution of heart rate variability indices in the frequency domain. Ptot: total power of the spectrum (ms2 / Hz), LF: low frequency (ms2 / Hz), HF: high frequency (ms2 / Hz) – Data from the *AuBE cohort.*

HRV is a good indicator for detecting and monitoring a stress level related to labor and delivery for the full-term newborn. After birth, the autonomic balance changes significantly during the first day of life. The slight sympathetic predominance observed at birth decreases in a few days in favor of the parasympathetic system, whose reactivity quickly becomes efficient (23). The rapidity of the cholinergic response (in milliseconds) compared to the thousand times slower adrenergic response (in seconds) will facilitate the onset of sudden cardiac slowdowns in response to extrinsic (noise, pain) or intrinsic (gastrointestinal reflux) stress (24). The sympathovagal balance of this neonatal period, specific to each individual, will then slowly modulate during the first months of life in favor of the parasympathetic branch, which will gradually become predominant, as described in the longitudinal *AuBE* (Autonomic Baby Evaluation) cohort (Figure 3). During the first 2 years of life, the healthy child benefits from a significant gain in overall autonomic maturation and gradually reaches a new equilibrium, resulting in a predominant parasympathetic activity compared to the sympathetic activity and, therefore, a fast and fine regulation gain (10).

Therefore, we must consider that this essential balance for homeostasis and cardiorespiratory control closely depends not only on wakefulness (wakefulness, calm sleep, active sleep) but also on postnatal age (25).

## Impact on the Decision to Stop Cardiorespiratory Monitoring in Neonatal Care Units

In the neonatal unit, the decision to stop cardiorespiratory monitoring before discharge requires careful tracking of daily modulations of heart rate, bradycardias, and desaturations and understanding the intrinsic self-regulatory capacities of newborns, and so by extension to analyze the basal autonomic balance and the ANS reactivity.

When the corrected term is reached, the cholinergic response is very efficient and faster than the adrenergic response. This singularity implies a physiological increase in the number of daily cardiac slowdowns as the term approaches for premature children. What matters then is not to count the daily bradycardias but to have a certainty on the capacity for sympathetic self-regulation (response), which must not be deficient. In other words, when the baby approaches the theoretical term, this vagal predisposition should not be considered as a pathological element. Conversely, a lack of orthosympathetic responsiveness could increase the risk of an inadequate cardiorespiratory response after internal or environmental stress. This lack of sympathetic response would increase the risk of Sudden Infant Death Syndrome (SIDS), especially in the premature population (26, 27).

In clinical practice, the occurrence of sinus bradycardias in a child who did not have it before may be the first symptom of a new problem and requires careful clinical examination. But when the baby approaches the theoretical term, a reflection on the capacity of autonomic self-regulation of the heart and respiratory rate should make it possible to safely stop the cardiorespiratory monitor in the vast majority of cases. Thus neonatal bradycardias do not justify continuing monitoring if, although numerous, they remain isolated, asymptomatic, brief (< 10 s), not deep (> 80 bpm), and followed by rapid cardiac acceleration testifying to an adapted sympathetic response. A complimentary assessment of newborns’ autonomic “capital” and their “responsiveness” makes it possible to identify children with a high potential for life-threatening event, who alone should benefit from cardiorespiratory monitoring at home (28–30).

This careful observation of the heart rate variability and complexity of respiratory rhythms, either in real-time or from a 24-hour cardiac Holter monitor, should become a valuable tool for considering autonomic control for neonatologists.

## Autonomous Maturation and Life-Threatening Events

Autonomic imbalance in the first few months may involve inappropriate cardiorespiratory responses after internal or environmental stress (31–35).

The neonatologist’s search for a congenital or acquired autonomic deregulation state as an objective risk factor for severe life-threatening event or unexpected infant death syndrom (28) should be a constant concern. In the SIDS triple risk model involving vulnerable children, exogenous stress, and critical development period, the cardiorespiratory autonomic control immaturity and abnormal arousal responses are predominant (33, 34). In an epidemiological survey of 20,000 children, Kato et al. have shown an association between central abnormalities of the cardiorespiratory response on awakening and life-threatening events and sudden death (34). In prematurity, Lucchini et al. showed a perfect correlation between the different experimental conditions of sleep-wake or prone and the multiparametric indices of HRV (30). Finally, the recent review by R. Horne (35) considers the association between cardiovascular control during infant sleep and the various components of the triple risk of SIDS, including maternal smoking.

Cardiorespiratory modulations during awakening periods are neurophysiologically mediated by the cortico-hypothalamic pathways and the cardiorespiratory nuclei of the brainstem (solitary tract, ambiguous nucleus, dorsal pneumogastric nerve). The molecular contribution of cardiorespiratory control inhibitory neurotransmitters such as GABA (γ-aminobutyric acid), adenosine, serotonin, endorphins, and prostaglandins in the genesis of apnea and bradycardia (36, 37) has been proposed in SIDS patients in particular with the identification of an abnormal serotoninergic response in the bulb and the arcuate nucleus of the hypothalamus, possibly due to genetic polymorphisms (38–40). Livolsi et al. reported overexpression of muscarinic M2 receptors in the brain, serum, and heart; and an increase in the enzymatic activity of acetylcholinesterase in case of severe life-threatening event or SIDS (41, 42).

All of these neurobiological considerations converge toward autonomic dysfunction as a preponderant element in the occurrence of SIDS.

## Acquired Dysautonomia in Neonatology

Studying the autonomic status of the child also has a predictive potential in many clinical situations frequent in the neonatal period, such as sepsis (43), anoxia (44), retinopathy of prematurity (45), and growth deficit (46). The pathogenic link between acute inflammation and dysautonomia during the neonatal period deserves to be refined even if it has been shown in case of chronic inflammatory diseases or diabetes (47, 48) an impairment of autonomic control and an increased risk of cardiovascular disease.

As part of routine care, analysis of HRV assessed in a non-linear domain could be of interest to predict extubation failure in very low birth weight premature infants (49).

It should also be remembered in a full-term neonatal model without pulmonary disease that in the case of non-invasive ventilation, the application of a continuous positive nasal pressure modifies the heart and respiratory rate variability by reducing the parasympathetic efferent activity without change in sympathetic efferent activity (50).

## Conclusion

The main interest of an HRV analysis from continuous monitoring is to obtain an objective reflection of the intrinsic capacity of the newborn to achieve perfect cardiorespiratory self-regulation. A state of congenital or acquired dysautonomia could be a central prerequisite for the occurrence of deleterious and life-threatening events.

The challenge of a real-time HRV assessment must be continued and complemented by clinical studies. It should make it possible to better target children at risk of SIDS.

## Data Availability Statement

The original contributions presented in the study are included in the article, further inquiries can be directed to the corresponding author.

## Author Contributions

HP conceptualized and wrote the article. PF, VP, and AG participated in certain studies described in the review, and refined the final manuscript with regard to recent data from the literature. All authors contributed to the article and approved the submitted version.

## Conflict of Interest

The authors declare that the research was conducted in the absence of any commercial or financial relationships that could be construed as a potential conflict of interest.

## Publisher’s Note

All claims expressed in this article are solely those of the authors and do not necessarily represent those of their affiliated organizations, or those of the publisher, the editors and the reviewers. Any product that may be evaluated in this article, or claim that may be made by its manufacturer, is not guaranteed or endorsed by the publisher.
